# Fast-Setting Concrete for Repairing Cement Concrete Pavement

**DOI:** 10.3390/ma16175909

**Published:** 2023-08-29

**Authors:** Tomasz Rudnicki, Przemysław Stałowski

**Affiliations:** 1 Faculty of Civil Engineering and Geodesy, Military University of Technology, 2 Kaliskiego St., 00-908 Warsaw, Poland; 2 Faculty of Civil and Environmental Engineering and Architecture, Bydgoszcz University of Science and Technology, 85-796 Bydgoszcz, Poland; przemyslaw.stalowski@pbs.edu.pl

**Keywords:** fast-setting concrete, compressive strength, pavement repair, industrial validation, real testing conditions

## Abstract

The paper presents the results of laboratory tests and the complete results of the implementation of the Sprint fast-setting concrete technology used during the reinstallation of the concrete pavement of the DK50 road (the Młodzieszyn bypass). The problem of concrete pavement repair is related to economic and social costs and, above all, to long repair time. After an extensive analysis of various technologies, the authors created a dedicated solution, which, based on commonly existing materials, allows for a very quick repair of the existing pavement. The obtained properties of the fast-setting concrete allow the maintenance of rheological parameters for 2 h from its production and to install the mixture of the consistency of class S3/S4, while the obtained compressive strength exceeds 25 MPa within 6 h of installation. The concrete parameters obtained after 24 h show a strength exceeding 40 MPa, and after 28 days—exceeding 60 MPa. The tensile strength at shattering exceeds 5 MPa, while the tensile strength at flexural strength exceeds 7 MPa. In addition, all parameters of the fast-setting concrete meet the highest requirements currently set for newly built concrete pavement loaded by very heavy traffic. The most important parameter is the possibility to shorten the repair time of concrete pavement to one day, significantly reducing the social costs associated with closing a single lane or the entire road. An important element of the presented technology is the possibility of producing the mixture at a stationary concrete batching plant, and in the future—with the use of bags for patching potholes in road pavement.

## 1. Introduction

**Figure 1:**
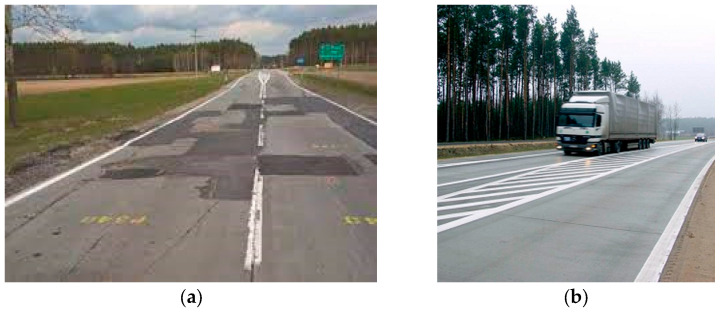
View of the concrete pavement before the renovation (**a**), and after the renovation (**b**) with the use of quick-setting concrete [GDDKiA archive].

In the last 10 years, in Poland, we have observed a rapid increase in the number of roads built in cement concrete technology, resulting from the fact that the main routes of motorways and expressways have been completed. The rapidly expanding road network requires preparation for possible repairs of the surface, which must be made under traffic within a certain operational time, minimizing difficulties for road users [1]. The available technologies for the repair of cement concrete pavements indicate limited possibilities of obtaining quick durability and cause great difficulties related to the exclusion of a lane or the entire roadway for up to 28 days. Therefore, an attempt was made to create fast-setting concrete that would allow for shortening the repair time of the concrete surface to 24 h, minimizing the inconvenience for users. The aim of the work of the Lafarge technology and development team was to create a technological solution that would allow for a quick replacement of the concrete slab and achieve a minimum of 20 MPa after 8 h from placing the concrete mix in order to reduce the traffic nuisance of users and the road administrator. The concrete mix for quick repair was developed on the basis of generally available materials with the possibility of its production both at stationary nodes and the production of dry mix in a bag. A very important feature of the mixture, on which work was carried out in the laboratory and tests at the production center, concerned maintaining workability in consistency class S3/S4 for 2 h. Fast-setting concrete, apart from meeting the requirements for obtaining very quick compressive strength, must be characterized by durability and resistance to frost and de-icing agents. An important element in the selection of the appropriate concrete pavement repair technology is the knowledge and proper identification of the causes of damage as part of the diagnostics of the exploited pavement [2,3,4,5]. The Highway Agency and other road infrastructure managers need quick repairs and require repairs to be completed within six or 8 h of the night so that the lanes can reopen the next morning to avoid disruption to road users [6]. The search for a fast-hardening concrete with high early strength that could be used to quickly repair the decks with minimal disruption to traffic became a priority [7]. For this reason, a new fast-curing HSFRC has been proposed, which is manufactured using sulfoaluminate cement (SC) instead of common Portland cement [7]. This type of concrete can be made on the actual construction site with rapid construction and is suitable for quick repair of various road and bridge surfaces. It can effectively solve the current problems of prolonged traffic closure, low traffic capacity and economic losses due to insufficient service life of repair materials when repairing the road surface [8]. According to the literature, proper recognition of the causes of damage is necessary to use the appropriate composition of the concrete mix, appropriate for a given solution [4]. Designing quick-setting concrete begins with the proper selection of binders, aggregates, and chemical additives in order to meet the required parameters of the concrete mix. In the case of surface repairs, fillers or mortars based on chemical additives, usually polymeric additives, can be used [3]. The type of repair material used in this case of the described fast-setting concrete depends not only on its composition, but also on the place of application. All environmental factors should be taken into account as they directly affect the effectiveness of the repair [2]. Damage to concrete pavements can be divided into superficial and structural. This division is used depending on the place of repair and destination [6]. The very composition of the binder in quick-setting concrete is important because, for example, a bellite-based binder cannot be used everywhere, because it is a mineral that increases the tightness of the mix. This can be successfully used in the case of surface repairs [7]. In the case of roads, it is important that the repaired element is quickly approved for use. This is not only an economic aspect but also a utility one. In other conditions, mixtures with a composition allowing good thin-layer bonding are used. [8]. More specifically, in the case of surface damage, these are primarily defects and chips of concrete, which are most often caused by the crushing of aggregates, micro-cracks in concrete and a network of cracks [9,10,11,12,13]. Another phenomenon is the peeling and abrasion of concrete, which occurs in the case of, for example, excessive drying of the surface during implementation or improper subsequent care [14]. Structural damage, on the other hand, includes keying expansion boards, damage to the expansion joints themselves, heaves under the pavement layer in the case of using the wrong foundation and fatigue cracks [15]. Repairing such a concrete surface is not difficult to implement and there are many ways. The repair process is conditioned by the condition of the surface and the type of damage, and it is the proper diagnosis, knowledge of the damage, and the current condition of the entire road that are the most important elements when carrying out repairs. Methods of repairing a concrete pavement are also divided into surface and structural ones [16]. In the case of the former, repairs consist mainly of grinding, smoothing or imparting texture to concrete by, for example, grinding [1,2], or supplementing small defects with filling or sealing compounds. Structural repairs are already more time consuming and require more work, as well as specialized equipment. In addition to surface repairs, e.g., patches, one such example may be the injection of cement grout under the slab, when the slabs are unevenly level or key in relation to each other. In the case of damage to the concrete dilatation, the repair consists of milling the expansion joint and filling it with a suitable sealing compound, or in completely reconstructing such a gap. However, the most time-consuming process is the replacement of slabs in the case of concrete fatigue cracks [5]. This method consists of dismantling the existing concrete surface, cleaning the area after demolition, sometimes additional dowels or reinforcing anchors must be made in order to connect adjacent slabs, and only then filling the loss with fresh concrete mix. Demolition and the repair itself can be done in a very short time; the biggest inconvenience is the later waiting for the release of such a repaired layer for traffic. Another type of concrete surface is concrete made in the rolling technology. The repair method in this case must be selected so as to maintain the homogeneity of the macrotexture, which affects the driving conditions [14]. As a rule, the technology of making a rolled surface is characterized by additional economic values. Therefore, it is important to consider not only qualitative but also economic aspects when selecting concrete mix components [13]. Taking into account, for example, airport pavements, which are also subject to damage, the time of shutdown of such a runway during repair is important [15]. In the case of concrete maturing, it is technologically required that the repair site be out of use for a minimum of 7 days, and optionally up to 28 days—i.e., the full cement hydration process. The method to speed up this process is the use of quick-setting or quick-setting concrete, which reaches its nominal strength within one day and not during the standard 28-day curing period. When designing a quick-setting concrete mix, materials should be used that can reduce the carbon footprint at the production stage and should be characterized by high durability allowing the use of such a surface over a long period of operation [17]. In addition, as previously mentioned, the amount and type of aggregate and binder should be selected in such a way as to obtain a similar or the same macrotexture of the surface at the repair site [18]. There are solutions for the production and use of ultra-high-performance concretes [19], but it should be adapted to the previously mentioned—place of repair, type of damage, and selection of repair technology. The opportunity to test the technology of quick-setting concrete appeared as a result of Lafarge’s cooperation with OAT sp. z o.o. during the reconstruction of the concrete surface of the national road No. 50 in the village of Młodzieszyna. During the execution of this task, the general contractor, OAT, was able to test both the possibility of replacing concrete slabs with ready-made prefabricated ones, as well as to carry out repairs under traffic with quick-setting concrete. The concrete surface of the Młodzieszyn bypass was subjected to cyclical loads, especially heavy vehicles, and resistance to temperature differences and frost for many years. View of the surface before renovation in Figure 1:

On the analyzed section of 2.4 km of the national road No. 50 of the Młodzieszyn bypass, work was carried out by a consortium of OAT and SAT companies in the period April–September 2019, under constant traffic in the field of repairing the concrete pavement by replacing slabs, levelling them, sealing expansion joints, roughening, and utilizing roadside reinforcements.

## 2. Materials and Methods

The selection of the type of materials for the design of Sprint 8 quick-setting concrete was based on the requirements of the technical specification [20]. CEM I 52.5 R cement (the characteristics of the cement are presented in Table 1), granite aggregate with a fraction of 2/8 and 8/16 mm, and sand with a fraction of 0/2 mm were used in the tests. In accordance with the requirements of the contract, a surface concrete of class C40/50 with a water/cement ratio below 0.40 was designed. The most important requirement for the high-speed concrete was to obtain a very high early strength (after 8 h > 20 MPa) and to maintain the workability of the mix for 2 h from production to incorporation into the pavement. In connection with the above, 3 types of chemical admixtures with a fluidizing effect and accelerating the setting of cement were used [21]. During the test batches at the concrete mixing plant in Warsaw, the properties of the concrete mix were verified after 5 and 120 min after mixing the ingredients in order to simulate the rheology behavior during long transport to the construction site. In addition to the tests of the concrete mix, the main requirements for compressive strength were determined after 8 and 24 h, and the final ones after 28 days of curing in conditions in accordance with the standard [22,23].

In the process of designing the mineral mix, granite aggregate of 2/8 and 8/16 mm fractions with a density of 2.65, and 0/2 mm sand with a density of 2.64 [g/cm^3^] was used. The design of the concrete mix began with determining the proportions between the aggregate of fractions 2/8 and 8/16 and sand in order to obtain the maximum saturation of the aggregate and the minimum free space between the grains. Table 2 below shows the grain size of the used granite aggregate 8/16 mm, granite aggregate 2/8, and sand 0/2 mm, and Table 2 shows the proportions of the mix composition.

In order to eliminate the possible risk of alkali–silica reaction (ASR), tests were carried out to determine the reactivity of the aggregate. The category of alkaline reactivity of the aggregates was determined on the basis of elongation measurements of mortar and concrete samples (direct methods), performed according to the PB/2/18 (long-term 365 days) or PB/1/18 (short-term 14 days) test procedures, respectively. Accelerated elongation testing of mortar samples according to PB/1/18 is carried out separately for fine aggregate 0/2 mm and coarse aggregate of fraction 2/8 and 8/16 mm. The R0 reactivity category is assigned to the aggregate if the elongation of the samples after 14 days of immersion of the mortar bars in a 1 M NaOH solution at 80 °C is not greater than 0.10% (coarse aggregate) or 0.15% (fine aggregate) [1]. The long-term elongation test of concrete samples should be used to evaluate aggregates (for fine aggregate and for all fractions of coarse aggregate combined). If the elongation of concrete samples after 1 year does not exceed 0.04%, then the aggregate reactivity category is R0.

The symbols R0 and R1 denote the degree of reactivity of the aggregate: R0 not reactive and R1 potentially reactive. The following symbols are used in Table 3 and Table 4: L7, L28, and L91 mean linear changes in the length of the samples during the test after 7, 28, and 91 days of testing according to the procedure for the determination of alkaline reactivity described in [1,21]. The results are presented in Table 3 and Table 4:

The test results obtained in the field of fine aggregate 0/2 mm and granite aggregate 2/8 and 8/16 mm meet the requirements of the test procedures and the aggregates have been classified as R0, i.e., non-reactive.

The next step was the appropriate selection of cement to ensure high early compressive strength. The key stage was the selection of chemical admixtures that ensured maintaining the liquid consistency for 2 h and obtaining a minimum of 20 MPa after 8 h.

The detailed composition of the designed recipes is presented in Table 5:

As part of the experimental research carried out in the central laboratory in Warsaw, over 300 samples were prepared and tested in order to determine the required properties. As part of the quality control of the delivered quick-setting concrete for the implementation of the investment of the General Directorate for National Roads and Motorways [24] for the task of redevelopment of the Młodzieszyn bypass into DK50, over 160 samples were prepared and tested, which were formed and stored during curing in accordance with the requirements of the PN-EN 12390-2 standard [23]. The detailed scope of testing the concrete mix and concrete is described in point 2.2 of the methods.

### Methods

Compressive strength tests were performed according to PN-EN 12390-3 [25] after 8 h, 24 h, and 28 days; concrete tensile strength according to PN-EN 12390-6 [26] and flexural strength according to PN-EN 12390-5 were performed [27] after 28 days of puberty. In the second part of the tests, the frost resistance was determined according to PN-B-06265 [28] and resistance to de-icing agents according to PKN-CEN/TS EN 12390-9 [29]. The frost resistance test was performed for 12 samples for each designed recipe. The components and their proportions in the concrete mix have been designed in accordance with the requirements for pavement concrete according to D-05.03.04 cement concrete pavement [20] and the catalog of typical rigid pavement structures [24] presented in Table 6 below:

## 3. Results

### 3.1. Compressive Strength

**Figure 2:**
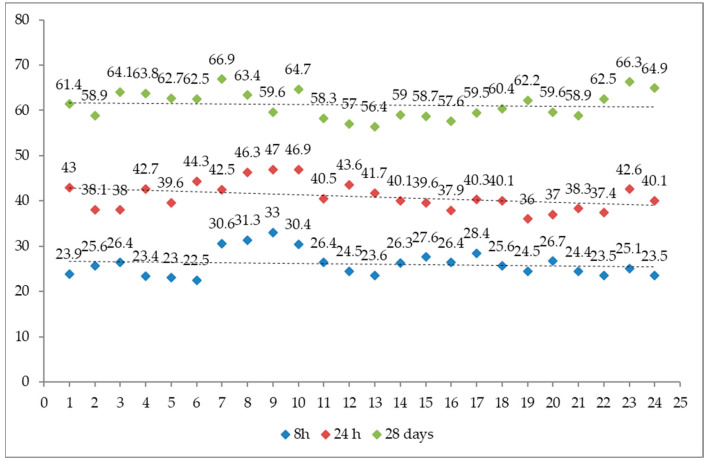
Compressive strength after 8 and 24 h and after 28 days.

The compressive strength was determined in accordance with the PN-EN 12390-3 standard after 8 and 24 h and after 28 days of concrete curing. As part of the compressive strength assessment, more than 150 samples were tested in accordance with the requirements of the EN 12390-2 standard. The test results are shown in Figure 2.

When analyzing the results of the quick-setting concrete compressive strength test, it should be noted that the most important requirement, i.e., a minimum of 20 MPa after 8 h, was met because the average result was 27.48 ± 3.6 MPa. The results of the compressive strength after 24 h are 42.77 ± 2.5 MPa, which, in the authors’ opinion, allows the road pavement to be approved for traffic. The compressive strength was also assessed after 28 days in accordance with the requirements of the GDDKiA technical specification [20,24], obtaining 63.88 ± 3.1 MPa.

### 3.2. Flexural Strength and Tensile Strength of Concrete

Flexural strength was determined in accordance with PN-EN 12390-5 after 28 days of curing. A total of 12 samples were prepared for the test, in accordance with the requirements of the EN 12390-2 standard. The tensile strength was determined in accordance with PN-EN 12390-6 for 15 samples in accordance with the requirements of EN 12390-2. The test results are presented in Table 7.

The flexural strength results obtained for the Sprint 8 quick-setting concrete met the requirements for concrete pavements for roads with very heavy traffic KR7, as they reached a minimum of 5.5 MPa for tensile strength and exceeded 3.7 MPa in the tensile strength test. Analyzing the obtained results, we can see that the average flexural strength obtained was 7.9 ± 0.7 MPa and the average tensile strength was 5.1 ± 0.2 MPa.

### 3.3. Determination of Frost Resistance after 150 Cycles

Concrete frost resistance was tested in accordance with PN-B-06250:1988 [28] using the standard method, procedure for F150 freezing and thawing cycles. From each of the tested mixtures, 12 cubes of 100 mm were formed. The produced specimens were then immersed in water for 28 days. Six samples of each blend were then weighed and subjected to 150 freeze–thaw cycles. The remaining six samples were left immersed in water as reference samples. After 150 freeze–thaw cycles, the samples were weighed and visually inspected. The compressive strength of all samples was determined as the last step in the test procedure. According to the frost resistance criteria PN-B-06250:1988 [28], the weight variation cannot exceed 5%, and the loss of compressive strength must not exceed 20% in relation to samples made of the same mixture that have not been subjected to cyclic freezing and thawing. Moreover, the samples after testing must not show any cracks. The test results are summarized in Table 8 below:

When analyzing the results of compressive strength tests obtained in the frost resistance test, it should be noted that the decrease in compressive strength after 150 cycles of freezing and thawing is only 2.3%. The tested samples of high-speed concrete built into the road surface met the condition required in the technical specification, because the decrease in compressive strength was less than 20% and the weight loss of the samples was less than 5%.

### 3.4. Determination of Resistance to Freezing and Thawing in the Presence of De-Icing Salts

Resistance to freezing and thawing in the presence of de-icing salts was carried out in accordance with the procedure described in EN 12.390 PKN-CEN/TS 12390-9 [29]. Sealed and protected samples were subjected to freeze–thaw cycles in 3% NaCl according to the procedure. The weight of the shelled material was determined after twenty-eight and fifty-six cycles for four samples. The average result of the three samples is shown in Table 9.

During the evaluation, it should be noted that for each of the samples, the weight loss of a single sample was less than 1.5 kg/m^2^ and the average weight loss determined after 28 and 56 test cycles was less than 1.0 kg/m^2^. The ratio of weight loss after 56 days to weight loss after 28 days was less than the required result 2. The freeze–thaw resistance values obtained in the presence of de-icing salts meet the requirements of the FT2 category for freeze–thaw specified in EN 13877-2:2013-08 [30].

### 3.5. Technical and Economic Analysis of the Use of Fast-Setting Concrete

In order to perform a technical and economic analysis of the comparison of concrete pavement repair technologies, two technologies (traditional concrete and fast-setting concrete) were compared. The performed analysis consists of calculations of both road management costs related to the renovation and social costs of users and environmental costs.

#### 3.5.1. Road Operator Costs

The basis for calculating the costs of the road administrator related to the renovation of the concrete pavement were the analyzed costs of concrete production, costs of technological facilities and labor, costs of concrete curing in the analyzed period, costs of occupying the right-of-way and costs of temporary traffic organization for the purposes of the works. In order to compare the costs of replacing slabs in the analyzed technologies, i.e., from traditional concrete pavement and quick-setting concrete, the following rates were adopted:

- The area of occupation of the surface for the replacement of a single slab, taking into account the space needed for technological service, including machine settings—4.5 m × 30 m = 135 m^2^;
- Occupation of the roadway to a width of 3.5 m, traffic lane + 0.5 m, marking = 4.0 m

4.0 m/8.0 m = 56%—the rate for occupying the right-of-way when the roadway is occupied more than 50%—pln 10 m^2^/day. For the purposes of further analysis we assume, therefore, 135 m^2^ × pln 10/m^2^—pln 1350/day;

- Costs of maintenance of the implemented temporary traffic organization—pln 500/day;
- Cost of technological facilities and labor—pln 4000/day;
- Costs of concrete care—pln 500/day;
- Costs of traditional surface concrete—pln 500/m^3^;
- Fast-setting concrete delivered from an external manufacturer—pln 3000/m^3^.

Calculations of the costs of replacing one slab of concrete pavement were made assuming that the slab has dimensions of 4.0 m × 6.0 m and a thickness of 0.25 m; i.e., 6.0 m^3^ of C35/45 cement concrete is needed to replace one slab.

A summary of the road manager’s costs of replacing one slab of concrete pavement is presented in Table 10:

When analyzing the costs of replacing one concrete slab, it should be noted that when comparing only the purchase cost of concrete, we obtain a much higher price for quick-setting concrete. However, if we analyze all the costs, such as road lane occupation, technical service costs of temporary traffic organization, maintenance costs, we can clearly see that the total cost of renovation using a traditional concrete mix is pln 75,500 and is four times higher than the comparable quick-setting concrete mix. However, the social costs of users and environmental costs must be considered in order to fully evaluate the cost comparison of high speed concrete technologies.

#### 3.5.2. Costs and Benefits of Road Users and the Environment

A very important, but often neglected, element of the analysis of the life cycle costs of concrete pavements are the costs of road users and the costs of the natural environment. For this purpose, the Manual for the Assessment of Economic Efficiency of Road and Bridge Projects of the Road and Bridge Research Institute in Warsaw was used. The economic cost categories of refurbishment are calculated on the basis of four main cost categories:

- Cost of operating vehicles based on technical data: type of vehicle (SO, SD, SCb, SCp, or A), topography (flat, undulating, or mountainous), road function (normal traffic—generally accessible, fast traffic—motorway, or express road), technical condition of the surface according to SOSN (A, B, C, or D), and travel speed of the motor vehicle;
- Cost of time for road infrastructure users in passenger transport and goods;
- The cost of road accidents and casualties;
- The cost of emission of toxic exhaust components.

In order to determine the costs of users and the environment, in accordance with the results of the GPR 2020/2021, data on the average daily annual traffic (SDRR) was determined at the measurement point No. 10906 DK50 on the section from km 66 + 010 to km 78 + 725. On this basis, the SDR for heavy vehicles and the SDR for passenger vehicles were determined. Detailed calculations as well as coefficients and their values used for further calculations were used in accordance with the instructions for assessing the economic effectiveness of road and bridge projects, IBDiM, and economic conditions for the operation of passenger regional transport in Poland according to the following formulas:

○ Vehicle operating costs:

(1) $$K_{e}=\sum_{j=1}^{5}k_{ej}\left(V_{pdrj},T,S\right)\cdot 365\cdot ({SDR}_{j}\cdot L).$$

According to the Formula (1), the difference between the cost of operation at a speed of V = 90 km/h and the cost of operation at a speed limited to V = 40 km/h was calculated. The obtained result is the real cost incurred by users, related to the operation of vehicles during repair works:

- Renovation in the traditional concrete technology, K_e_ = pln 96,525;
- Renovation in the technology of fast-setting concrete, K_e_ = pln 3217.

In the course of carrying out works in the traditional concrete technology for a period of 30 days, the costs of operating vehicles in connection with the speed limit from 90 km/h to 40 km/h incurred by traffic users amount to pln 96,525; in the case of using high-speed concrete, they amount to only pln 3217 and constitute only 3.3% of the costs incurred by drivers in the traditional surface concrete technology.

○ Time costs of road infrastructure users in passenger transport:

(2) $$K_{c}=L\cdot \sum_{j=1}^{2}\frac{k_{cj}}{V_{pdrj}}\cdot 365\cdot {SDR}_{j}.$$

According to the Formula (2), the difference in cost between the standard cost at a speed of V = 90 km/h and the cost at a speed limited to V = 40 km/h was calculated; the result is the difference, which is the real cost incurred by users related to the time of road infrastructure users in passenger transport:

- Renovation in traditional concrete technology, K_c_ = pln 11,266;
- Renovation in the technology of fast-setting concrete, K_c_ = pln 376.

The time costs of road infrastructure users in passenger and freight transport due to the speed limit from 90 km/h to 40 km/h incurred by traffic users amount to pln 11,266; in the case of using high-speed concrete, they amount to only pln 376 and account for only 3.3% of the costs incurred by drivers in the traditional surface concrete technology.

○ Costs of road accidents and victims of road infrastructure users:

(3) $$K_{w}=L\cdot w_{wa}\cdot k_{w}\cdot 365\cdot \sum_{j=1}^{5}\left(\frac{{SDR}_{j}}{1000000}\right).$$

Analyzing the risk of road accidents on the section covered by the study, i.e., 1 km, taking into account the average costs of a road accident and accident victims, we obtain costs estimated on the basis of the risk of an event occurring during the works in individual technologies, taking into account the time of their implementation:

- Renovation in the traditional concrete technology, K_w_ = pln 174,135;
- Renovation in the technology of fast-setting concrete, K_w_ = pln 5804.

The risk of an accident in the long term of the works is significantly higher than in the case of one-day works, the risk of costs incurred in the case of traditional surface concrete is estimated at pln 174,155, and in the case of high-speed concrete technology only pln 5804, which proves a significant reduction in the risk of an accident and minimizes social costs.

○ Environmental pollution costs of road infrastructure users:

(4) $$K_{s}=\sum_{j=1}^{5}365\cdot L\cdot k_{s,i}\cdot {SDR}_{j}.$$

Substituting the individual coefficients into the Formula (4), and in particular the unit costs of environmental pollution in relation to the type of vehicle and the type of terrain on which the given vehicle is moving, we obtain the costs of environmental pollution of road infrastructure users:

- Renovation in traditional concrete technology, K_s_ = pln 36,985;
- Renovation in the technology of fast-setting concrete, K_s_ = pln 1233.

The costs of environmental pollution mainly consist of air pollution with nitrogen oxides NO, carbon dioxide CO_2_ emissions, and road noise. The greatest nuisance for the environment is generated by traffic restrictions related to the execution of works, in the traditional surface concrete technology they amount to pln 36,985, and in the quick-setting concrete technology, only pln 1233, which is 3.3% of the costs compared to the traditional concrete technology. The costs of users and the environment are hidden costs, very often they are not directly analyzed and generated for the investor and, thus, are most often omitted in the process of determining the cost of renovation. In order to show the actual costs incurred by the road user and the environment, a tabular summary was made, taking into account the conditions prevailing at the time before the renovation, i.e., for the travel speed of V = 90 km/h and with the traffic limited to the speed of V = 40 km/h. Such a comparison of the costs and possible benefits of reducing the repair time from 30 to 1 day is presented in Table 11:

In the case of the traditional surface concrete technology, the costs of users and the environment are pln 318,911; in the case of the use of high-speed concrete technology, the costs are only pln 10,630. Analyzing the obtained results, it can be concluded that the greatest impact on such a large difference is the costs of road accidents, which account for 55% of the costs, and the costs of vehicle operation, which account for 30% of the costs of users and the environment.

However, the most important conclusion from the analysis is the possibility of generating savings for users and the environment through the use of quick-setting concrete, amounting to pln 308,281.

### 3.6. Discussion

The aim of the research project was to design and implement a quick-setting concrete that meets the requirements of the contracting authority, i.e., to achieve a minimum of 20 MPa after 8 h and workability up to 2 h. During the renovation of the Młodzieszyn bypass, an average of 24.5 MPa was achieved after 8 h and over 42.8 MPa after 24 h of construction. In addition, the concrete mix maintained the consistency of S4 after 2 h of transport to the construction site, which greatly facilitated the placement of concrete. Analyzing the obtained concrete compressive parameters, a thesis can be put forward that after one day such a surface can be loaded with heavy traffic because the obtained compressive strength exceeded 42.8 MPa. The results of testing such features as flexural tensile strength and splitting significantly exceeded the requirements and amounted to 7.9 MPa and 5.1 MPa, respectively. In accordance with the requirements for surface concretes, frost resistance was determined after 150 cycles of alternating freezing and thawing to obtain a decrease in compressive strength of 2.3% and a loss of mass of 0.29%. It is worth noting that none of the tested samples showed cracks or damage after the frost resistance test. Such results testify to the very high quality and durability of the discussed concrete. In addition, the experimentally determined resistance to freezing and thawing in the presence of salt (NaCl) proves its resistance to combined exposure to frost and de-icing salt. The fast-setting concrete offered by Lafarge met all the requirements contained in the technical specification and can be successfully used as an alternative solution for the repair or partial replacement of concrete pavement for very heavy traffic. The most important advantage of this solution is shortening the repair time to 24 h and reducing the nuisance for users by lowering economic and social costs. When analyzing the costs of renovation in traditional technology and quick-setting concrete, the full costs of the road infrastructure manager should be taken into account. These costs should include not only the costs of the concrete mix but also the costs of occupying the right-of-way, maintenance costs, costs of technological facilities, and maintenance. After making full calculations, it turns out that the use of fast-setting concrete is economically justified. A very important element of the analysis is the possibility of generating additional economic benefits for road users and the natural environment, which in the analyzed case amount to over pln 308,000.

## 4. Conclusions

Based on the research and analysis of high-speed concrete carried out by the authors, the following conclusions can be drawn:

(1) The compressive strength was 27.5 MPa after 8 h and more than 42.8 MPa after 24 h, which means that this concrete meets the highest requirements for repair concretes.
(2) Values of frost resistance after 150 cycles of alternating freezing and thawing as well as determination of resistance to de-icing agents testify to very high quality and durability of concrete.
(3) The concrete mix tested during delivery and after 2 h of transport was characterized by high workability and the marked consistency was at the level of S3/S4.
(4) An example of the practical use of concrete during the renovation of the Młodzieszyn bypass allows to shorten the time of repair or replacement of the concrete surface to one day, minimizing economic and social costs for road users and the road administrator.
(5) In further research plans, the authors plan further research on the possibility of shortening the time of repair or replacement of the concrete pavement through the use of new polymer admixtures and additives for concrete.

Future directions and limitations—The presented solution, apart from tests in the laboratory, was industrially implemented on the national road No. 50 during the renovation of the concrete pavement. Renovation of the road with a concrete surface was carried out for the General Directorate for National Roads and Motorways. The obtained test results of the built-in fast-setting concrete were positively assessed in terms of quality and test results obtained. Further modifications of the concrete composition are planned in the near future, aimed at obtaining early compressive strength after 6 h, and not after 8 h as before.

## Figures and Tables

**Table 1:** Properties of cement CEM I 52.5 R provided by the manufacturer.

Property	Unit	Type of Cement
Water Lust	%	32.7
Beginning of cement setting	minutes	208
End of cement setting	minutes	266
Consistency in volume	mm	0.9
Specific surface	cm^2^/g	4612
Specific density	cm^3^/g	3.14
Compressive strength 1 day	MPa	28.7
Compressive strength 2 days	MPa	41.1
Compressive strength 28 days	MPa	65.8
SO_3_	%	3.1
eqNa_2_O	%	0.82
Cl^−^	%	0.07

**Table 2:** Graining of materials.

Sieve [mm]	Screening by Volume [%]		
	Granit 8/16	Granit 2/8	Sand 0/2
16.000	1.7	0.0	0.0
8.000	96.3	1.2	0.0
4.000	2.0	59.7	0.0
2.000	0.0	39.1	2.5
1.000	0.0	0.0	15.9
0.500	0.0	0.0	25.5
0.250	0.0	0.0	43.4
0.125	0.0	0.0	11.9
0.000	0.0	0.0	0.8

**Table 3:** Determination of the ASR alkali–silica reaction for 0/2 mm aggregate.

Samples	Length Change over Time [Day]					
	L_7_	L_14_	L_28_	L_91_	L_182_	L_365_
1	−0.002	0.003	0.001	0.008	0.014	0.016
2	−0.004	0.003	0.001	0.009	0.012	0.015
3	−0.003	0.003	0.002	0.007	0.012	0.014
Average value	−0.003	0.003	0.001	0.008	0.013	0.015

**Table 4:** Determination of the alkali–silica ASR reaction for 2/8 and 8/16 mm aggregate.

Samples	Length Change over Time [Day]					
	L_7_	L_14_	L_28_	L_91_	L_182_	L_365_
1	0.000	−0.001	−0.002	0.006	0.018	0.027
2	−0.001	0.000	−0.003	0.006	0.016	0.019
3	−0.001	−0.001	−0.001	0.006	0.016	0.022
Average value	−0.001	−0.001	−0.002	0.006	0.017	0.023

**Table 5:** Composition of concrete mixtures.

Materials	Concrete Mix Compositions, by Volume %
CEM I 52.5 R	16.46
Water	15.31
Granit 2/8	23.81
Granit 8/16	19.51
Sand 0/2	22.82
PC 1	0.19
PC 2	0.10
PC 3	1.81

**Table 6:** Requirements for the concrete pavement for traffic categories KR5 ÷ KR7.

Properties of Pavement Concrete	Requirements	Test Method
Compressive strength class for traffic category KR5 ÷ KR7, not lower than:	C40/50	PN-EN 12390-3
Flexural strength of concrete for traffic category KR5 ÷ KR7, not lower than:	5.5 MPa	PN-EN 12390-5
Tensile strength of concrete when splitting for traffic category KR5 ÷ KR7, not lower than:	3.7 MPa	PN-EN 12390-6
Concrete frost resistance test F150:		
- Weight loss of the sample, not more than, % - Decrease in compressive strength, no more than, %	5% 20%	PN-B-06250

**Table 7:** Flexural strength and tensile strength of concrete.

Description of the Designation	Results	Average Result	Requirement
Flexural strength, MPa	7.2	7.9 ± 0.7	>5.5
	8.6		
	7.8		
Tensile strength, MPa	4.8	5.1 ± 0.2	>3.7
	5.3		
	5.1		

**Table 8:** Influence of cement type on frost resistance after 28 days of maturation.

Frost Resistance Test F150				
Description of the Designation	Average Result	R_c_ (MPa)	R_c150_ (MPa)	Requirement
Mean decrease of the strength of specimens ΔR, %	2.30%	62.7	66.3	<20%
		68.4	66.7	
		73.8	70.1	
Mass change of specimens subjected to cyclical freezing and thawing ΔG, %		2320	2314	<5%
	0.29%	2386	2378	
		2344	2337	

**Table 9:** Determination of resistance to freezing and thawing in the presence of de-icing salts.

Mass Loss (kg/m^2^)		Degree of Defect	
After 28 Cycles	After 56 Cycles	m_56_/m_28_	Requirement
0.024	0.030	0.80	<2.0
0.013	0.015	0.87	
0.028	0.033	0.84	
0.027	0.032	0.85	

**Table 10:** Costs of replacing one board.

Renovation Item	Time	Unit Price	Traditional Concrete	Fast-Setting Concrete
Cost of concrete	1	2000 × 6 m^3^		12,000
	30	500 × 6 m^3^	3000	
Roadway cost	1	135 m^2^ × 10 pln		1350
	30	135 m^2^ × 10 pln	40,500	
Costs of technical service of temporary traffic organization	1	500 pln/day		500
	30	500 pln/day	15,000	
Cost of technological facilities and labor	1	4000 pln/day		4000
	3	4000 pln/day	12,000	
Concrete curing costs	1	500 pln/day		500
	10	500 pln/day	5000	
Sum of costs			75,500	18,350

**Table 11:** Summary of user and environmental costs for selected technologies.

User and Environmental Costs	Traditional Road Concrete	Fast-Setting Concrete
Vehicle operating costs V = 40 km/h	811,170 pln	27,039 pln
Vehicle operating costs V = 90 km/h	714,645 pln	23,822 pln
Operating costs K_e_ V = 40 km/h—V = 90 km/h	96,525 pln	3217 pln
User time costs V = 40 km/h	20,279 pln	676 pln
User time costs V = 90 km/h	9013 pln	300 pln
User time costs K_c_ V = 40 km/h—V = 90 km/h	11,266 pln	375 pln
Costs of road accidents and casualties	174,135 pln	5804 pln
Environmental pollution costs	36,986 pln	1232 pln
User and environmental costs for V = 40 km/h	318,911 pln	10,630 pln
Savings compared to the use of traditional concrete	0 pln	308,281 pln

## Data Availability

Not applicable.
